# Probiotic Bacilli Inhibit *Salmonella* Biofilm Formation Without Killing Planktonic Cells

**DOI:** 10.3389/fmicb.2021.615328

**Published:** 2021-02-17

**Authors:** Mahtab Hassanpour Tazehabadi, Ammar Algburi, Igor V. Popov, Alexey M. Ermakov, Vladimir A. Chistyakov, Evgeniya V. Prazdnova, Richard Weeks, Michael L. Chikindas

**Affiliations:** ^1^Department of Biological Sciences, School of Environmental and Biological Sciences, Rutgers State University, New Brunswick, NJ, United States; ^2^Department of Biotechnology, College of Science, University of Diyala, Baqubah, Iraq; ^3^Department of Scholarship and Cultural Relation, Presidency of Diyala University, Baqubah, Iraq; ^4^Research Laboratory «Agrobiotechnology Center», Don State Technical University, Rostov-on-Don, Russia; ^5^Experimental Mutagenesis Laboratory, Southern Federal University, Rostov-on-Don, Russia; ^6^Health Promoting Naturals Laboratory, School of Environmental and Biological Sciences, Rutgers State University, New Brunswick, NJ, United States; ^7^I. M. Sechenov First Moscow State Medical University, Moscow, Russia

**Keywords:** probiotics, biofilm inhibition, *Salmonella*, poultry, *Bacillus*

## Abstract

Salmonellosis is a foodborne infection caused by *Salmonella.* Domestic poultry species are one of the main reservoirs of *Salmonella*, which causes the foodborne infection salmonellosis, and are responsible for many cases of animal-to-human transmission. Keeping backyard chickens is now a growing trend, increasing the frequency of direct contact with the flock and, by consequence, the incidence of *Salmonella* infections. *Bacillus subtilis* KATMIRA1933 and *Bacillus amyloliquefaciens* B-1895 are probiotic bacilli that produce the bacteriocins subtilosin A and subtilin, respectively. The antimicrobial activity of the two strains was determined against the reference strain *Micrococcus luteus* ATCC 10420. The cell-free supernatant of *B. subtilis* KATMIRA1933 inhibited biofilm formation by *Salmonella enterica* subsp. *enterica* serovar Hadar, *Salmonella enterica* subsp. *enterica* serovar Enteritidis phage type 4, and *Salmonella enterica* subsp. *enterica* serovar Thompson by 51.1, 48.3, and 56.9%, respectively. The cell-free supernatant of *B. amyloliquefaciens* B-1895 inhibited the biofilm formation of these *Salmonella* strains by 30.4, 28.6, and 35.5%, respectively. These findings suggest that the bacillus strains may have the potential to be used as probiotics and antibiotic alternatives for the control of *Salmonella* in poultry. The number of planktonic cells was unaffected by treatment with the cell-free supernatant. A co-culture of the *Salmonella* strains with either bacilli showed no signs of growth inhibition, suggesting that it might have been quorum sensing that is affected by the two *Bacillus* strains.

## Introduction

Salmonellae are pathogens in both humans and animals and are responsible for causing salmonellosis, which is most commonly (85%) a foodborne illness. However, infection due to animal contact and person-to-person transmission of the disease are also possible (Hung et al., 2017). There are approximately 1.2 million cases of non-typhoidal salmonellosis (NTS) in the United States each year (Dekker and Frank, 2015; Ajmera and Shabbir, 2020). Pathogenic salmonellae most often act on the gastrointestinal tract, where they are able to colonize and invade the mucosa of the small intestines and colon. The severity of the disease’s manifestation depends on the patients’ level of sensitivity to the pathogen and how virulent the particular serovar is^1^. NTS typically presents as gastroenteritis with diarrhea, fever, and abdominal cramps, and is generally mild, resolving without treatment within 1–4 days. However, in rare cases, *Salmonella* infection can progress to an invasive, extra-intestinal disease leading to bacteremia and focal systemic infections known as invasive NTS^2^ (Gal-Mor et al., 2014).

Upon ingestion, the stomach’s low pH (<3.5) is lethal to Salmonella and has a significant impact on the ability of an infectious dose to reach the intestines. Salmonellae also have varying degrees of constitutive and inducible acid resistance, providing some protection, while association with the ingested food matrix may further protect cells (Bertelloni et al., 2017). Additional host defenses include gastrointestinal proteases, defensins, and other innate and adaptive components of the immune system. The intestinal microflora also plays an important role in fighting against salmonellosis by producing antimicrobial substances such as short-chain fatty acids, bacteriocins, and bacteriocin-like inhibitory substances (BLIS) that are thought to be toxic to *Salmonella*. It is important to note that treatment with antibiotics may change the composition of the intestinal flora and make the host more susceptible to *Salmonella* infection (Foley et al., 2013).

*Salmonella* is a Gram-negative, facultative anaerobic, and flagellated bacterial genus containing two species, *S. enterica* and *S*. *bongori*, with more than 2,500 identified serovars. *S. enterica* subsp. *enterica* represents the majority of pathogenic salmonellae in humans and other warm-blooded species, accounting for 99.5% of all isolates (Dekker and Frank, 2015; Wang et al., 2019a). The most common hosts for animal-human transmission are livestock while the main sources of pathogenic *Salmonella* infecting humans are food products including eggs, egg products, and poultry meat (Chlebicz and Śliżewska, 2018; Akil and Ahmad, 2019). Contaminated foods are the main mode of transmission as *Salmonella* remains viable and can proliferate on meat and animal products that are not cooked or stored properly. In some cases, humans and animals may become chronic carriers of *Salmonella*, which can persist for years without obvious clinical signs of infection. The wide array of animal reservoirs, ease of transmission, and the prevalence asymptomatic carriers make salmonellosis an ongoing and critical public health issue. Additionally, the overuse of antibiotics in animals and humans has led to an increase in antibiotic resistant *Salmonella*. Antibiotic resistance reduces the effectiveness of current treatment strategies, and can extend the carriage time, causing patient to shed the bacteria in their feces over a longer timeframe^1^, increasing the spread and persistance of the illness.

The increased popularity of sustainably and locally produced food is now a major contributing factor to the spread of *Salmonella*. There is also a growing trend of keeping backyard chickens, often treating them as pets, greatly increasing the incidence of direct human contact with potential *Salmonella* carriers. Unfortunately, many are unaware of the regulatory guidelines that should be followed when raising chickens. This can result in poor hygiene when handling chickens and eggs, and may also lead to the improper use of antibiotics for the prevention and treatment of infectious diseases (McDonagh et al., 2019). The Centers for Disease Control and Prevention (CDC) has investigated several multistate outbreaks of *Salmonella* that were found to be linked to direct contact with backyard poultry^1^.

This evidence suggests that contact with poultry, no matter how clean or healthy they appear to be, can make humans sick, especially for children younger than five^1^. Salmonellosis is rare in poultry older than 2 months, and *Salmonella* infections are predominantly asymptomatic, furthering the pathogens spread. The 42°C body temperature of chicken is believed to reduce the expression of the *Salmonella* pathogenicity island 1 (SPI-1). However, colonization is still possible with only a small number of cells expressing SPI-1, and the proliferation of *Salmonella* within the intestines is still possible. Lowered expression of SPI-1 is likely one of the factors responsible for the asymptomatic persistence of *Salmonella* in the chicken GIT (Eade et al., 2019). Since the host chicken is asymptomatic, owners may assume that it is safe to be in close contact with the animal. As a result, the chances of transmission of salmonellosis from an infected chicken to humans are considerably increased (Nair et al., 2018).

Biofilm production is an important virulence factor in *Salmonella* that contributes to the persistence of *Salmonella* in the gut of humans and livestock following the initial infection (Fàbrega and Vila, 2013). Biofilms increase the chances of the microorganisms’ survival by inducing antimicrobial resistance, mechanical persistence, and the production of virulence factors (Giaouris et al., 2015). For a biofilm to successfully form and remain viable an effective cell-to-cell signaling system must be present. In bacteria, a range of specific and diffusible signaling molecules called autoinducers are produced in response to changes in cell density. Cells within a biofilm can detect and respond to these molecules and are therefore able to coordinate their activities. *Salmonella* spp. have quorum sensing systems mediated by three different classes of autoinducers: AI-1, AI-2, and AI-3. AI-1 has been shown to induce biofilm formation by *S. enteritidis* under anaerobic conditions, and an antagonistic structural analog of AI-2, patulin, has been demonstrated to inhibit biofilm formation by quorum quenching (Vijayababu et al., 2018). Quorum sensing inhibitors have also shown promise as potential biofilm dispersing agents, as reviewed by Brackman and Coenye (2014). Therefore, the disruption of various quorum sensing mechanisms may be a promising strategy for the control of biofilm formation and treatment of established biofilms (Giaouris et al., 2015).

Biofilms in poultry and other livestock serve as a source of contamination at nearly every step in the food processing pipeline. *Salmonella* shed from livestock can infect food workers and will also form biofilms on biotic and abiotic surfaces including eggs and meat, but also in processing plants, packaged foods, waste water, and eventually, both commercial and household environments (Lamas et al., 2018). *Salmonella* biofilms in the food processing pipeline may be controlled through a variety of measures, including proper hygiene standards and use of personal protective equipment for food workers, the use of antibiotics and other antimicrobials in livestock, and physical or chemical sterilization for food processing plants, kitchens, and storage areas (Lamas et al., 2018). However, these measures often fail to eradicate biofilms entirely, allowing for the continuous spread of *Salmonella*, which may increase the resistance of *Salmonella* to these treatments. As infected livestock are often the major initial source of *Salmonella* in other environments, it only makes sense to investigate new approaches for preventing *Salmonella* infection and the carrier-state in poultry.

As mentioned previously, an increase in the use of antibiotics can lead to antibiotic-resistance as well as disruption of the normal microbial flora, with around 100,000 infections due to antibiotic-resistant *Salmonella* alone each year (Nair et al., 2018). While salmonellosis typically resolves on its own without treatment, more serious cases may need intervention, which typically involves the use of antibiotic drugs. Common first line treatments for NTS in the United States include fluoroquinolones in adults and azithromycin in children. However, drug resistance and multidrug resistance in *Salmonella* is now common. For example, in a 2019 outbreak, the *Salmonella* Infantis strain identified was resistant to, “ciprofloxacin, ceftriaxone, or other antibiotics including ampicillin, chloramphenicol, fosfomycin, gentamicin, kanamycin, nalidixic acid, streptomycin, sulfisoxazole, trimethoprim-sulfamethoxazole, and tetracycline.^3^” In these cases, it is recommended to determine the appropriate antibiotic based on individual antimicrobial susceptibility testing, which is not always possible and can be costly and time consuming. In poultry, antimicrobial treatment for the control of *Salmonella* is rare and strongly discouraged, requiring veterinary oversight in the US, especially in the case of medically important antimicrobial drugs. In most cases, the negative disease status of flocks is maintained through the eradication of the entire flock if it is found to be harboring *Salmonella* (Agunos et al., 2012).

Therefore, scientists are now looking at alternative strategies for controlling and treating *Salmonella* infections. Newer strategies include prophylactic measures, such as the development of vaccines, as reviewed by Desin et al. (2013), or the use of probiotics, prebiotics, and their derivatives, such as fermentates, feed additives, and antimicrobial peptides. The use of probiotics and their derivatives in the control of pathogens and prevention of antimicrobial resistance is a promising alternative to traditional antibiotic treatments. The World Health Organization defines probiotics as “live microorganisms which when administered in adequate amounts confer a health benefit to the host.” In addition to their antimicrbioal potential, the presence of probiotics in poultry feed has been shown to improve overall growth and intestinal health, and provide other benefits related to meat and egg quality (Popova, 2017; Makarenko et al., 2019). Probiotics can also boost the immune system, stimulate endogenous enzymes, produce antimicrobial substances, and reduce the production of toxic substances by controlling the metabolic pathways for their synthesis. Antimicrobial substances produced by probiotics are also able to inhibit the production of toxins and the adhesion ability of some pathogens (Monteagudo-Mera et al., 2019).

Spore-forming bacteria as probiotics have become more popular in recent years. Spores are dormant bacterial structures that are highly resistant to hostile environmental conditions. Bacterial spores greatly increase the chance of survival when exposed to UV radiation, temperatures of 80–85°C, various solvents, hydrogen peroxide, and enzymes such as lysozyme. Spores possess notable advantages as probiotics, including storage at room temperature without losing viability, and the ability to safely pass through the gastric bactericidal barrier. When spores find themselves in a less hostile environment, such as the intestines, they germinate and resume vegetative cell growth (Cutting, 2011). *B. subtilis* are Gram-positive spore-forming bacteria, some of which are used as probiotics in chicken. Studies have shown that *B. subtilis* spores have the ability to germinate in the gastrointestinal tract of poultry (Cartman et al., 2008).

*B. subtilis* KATMIRA1933 is a strain isolated from a dairy product that has been shown to have probiotic capacities related to the production of bacteriocins, which are ribosomally-synthesized antimicrobial peptides. They often have a narrow range of activity, and often only target species that are closely related to the bacteriocin producer (Shelburne et al., 2007). Subtilosin A, which is a cyclic peptide, is one of the primary bacteriocins produced by *Bacillus subtilis*. It has been shown to have antimicrobial activity against a range of bacteria, including Gram-positive, Gram-negative (in the presence of chelating agents), aerobic, and anaerobic species (AlGburi et al., 2016). Subtilosin A is largely hydrophobic and is likely to act in part due to interactions with the hydrophobic portion of the phospholipid bilayer of the cell membrane. Subtilosin A also has a negatively charged region that remains exposed to the outside environment and may act on receptors on the membrane’s surface. The antimicrobial activity of subtilosin A against several pathogens, such as *Listeria monocytogenes*, *Gardnerella vaginalis*, and *Streptococcus agalactiae* has been reported (Nikiforova et al., 2016). *B. amyloliquefaciens* B-1895 is another probiotic with antimicrobial properties. *B. amyloliquefaciens* B-1895 was originally isolated from a soil sample and has been found to produce a variety of proteolytic enzymes. It also produces the antimicrobial peptide subtilin, which has been shown to have activity against the foodborne pathogen *Listeria monocytogenes* (Algburi et al., 2020b).

The purpose of this study was to investigate the effects of *B. subtilis* KATMIRA193s and *B. amyloliquefaciens* B-1895 and their cell-free supernatants (CFS) on the biofilm formation ability and planktonic cell viability of pathogenic *Salmonella* species. Both strains harbor the genes coding for antimicrobials (Karlyshev et al., 2014a,b) and are probiotic microorganisms promoting the health of poultry (Prazdnova et al., 2019).

## Materials and Methods

### Bacterial Culture and Growth Condition: *B. subtilis* KATMIRA1933 and *B. amyloliquefaciens* B-1895

Streak plates of *B. subtilis* KATMIRA1933 and *B. amyloliquefaciens* B-1895 were made using DeMan, Rogosa, and Sharpe agar (MRS, BD Difco, Franklin Lakes, NJ, United States) from frozen stock maintained in the Health Promoting Naturals Laboratory collection at −80°C. The plates were incubated aerobically at 37°C for 24 h. A single colony was picked from each plate and inoculated into MRS broth in 15 mL conical centrifuge tubes (Corning, Corning, NY, United States). The tubes were incubated at 37°C for 18 h and were tilted at 45 degrees in order to increase the surface area for maximum aeration. Two subsequent subcultures of 1:100 dilutions were made under the same conditions.

### Antibacterial Activity of Individual Colonies of *B. subtilis* KATMIRA1933 and *B. amyloliquefaciens* B-1895 Against *Micrococcus luteus* ATCC 10420

The reference strain *Micrococcus luteus* ATCC 10240 is commonly used for the testing of bacteriocins’ antimicrobial activity (Mauriello et al., 2005). The microorganism was obtained from the American Type Culture Collection (ATTC, Manassas, Virginia, United States). A subculture of *M. luteus* was streaked on MRS agar and incubated at 37°C for 24 h. Ten single colonies of *B. subtilis* KATMIRA1933 and *B. amyloliquefaciens* B-1895 were selected and then plated on two different sets of MRS agar to obtain replicate plates. The plates were incubated at 37°C for 18 h. One of the plates was treated with chloroform vapor in a biosafety cabinet (SG 400, The Baker Company, Sanford, Maine, United States) for 60 min to kill the cells. A piece of filter paper was placed on the lid of the petri dish and 2 mL of chloroform was added to it every 20 min, with the agar plate placed upside down above the lid during treatment. A soft agar overlay test was used to identify single colonies with the best production of antimicrobials, as determined by a comparison of the diameters of the observed zones of inhibition for each colony. Two milliliter of soft agar seeded with 100 μL of 10^6^ CFU/mL *M. luteus* ATCC 10240 was poured onto the chloroform-treated plate. The treated plates were incubated at 37°C for 24 h, and the untreated plates were kept at 4°C. The colony with the largest zone of inhibition was selected for each strain. The corresponding untreated colony was then streaked, and the same procedure was performed again. The experiment was repeated three times in order to obtain the best and most consistent antimicrobial producing colony for both strains. Frozen stocks (1:1 solution of 50% glycerol in water together with overnight growth in MRS broth) were made from the colonies for further experiments.

### *Salmonella* and Bacilli Cross Test

A cross-test was performed to assess the potential antagonistic activity of the two bacilli on *Salmonella* when grown on solid media. The cross test was performed according to Balouiri et al. with some modifications (Balouiri et al., 2016). Two vertical lines of 10^6^ CFU/mL *B. subtilis* KATMIRA1933 or *B. amyloliquefaciens* B-1895 were streaked 5 cm apart on a TSA plate using an inoculation loop (BD Difco, Franklin Lakes, NJ, United States), and the plates were left to dry for 10 min. Then, a horizontal line of 10^6^ CFU/mL *Salmonella* species (Thompson, Enteritidis phage type 4, and Hadar) was streaked perpendicular to the bacilli streaks by an inoculation loop. The plates were incubated at 37°C for 24 h. Then, they were kept in a biosafety cabinet at room temperature for an additional 3 days.

In a second experiment, two vertical lines of 10^6^ CFU/mL *B. subtilis* KATMIRA1933 or *B. amyloliquefaciens* B-1895 were streaked 5 cm apart on a TSA plate using an inoculation loop, and the plates were incubated at 37°C for 24 h. Then, the *Salmonella* species were streaked as in the previous experiment. The plates were incubated at 37°C for 24 h and were kept in the hood for an additional 3 days.

### *Salmonella* and Bacilli Co-culture

A co-culture experiment was performed to assess the potential antagonistic activity of the two bacilli on the viability of *Salmonella* when grown in liquid medium. Overnight cultures of *Salmonella* and bacilli were prepared. One hundred microliter of the overnight cultures was added to 9.9 mL of TSB in order to dilute the samples to 10^7^ CFU/mL. One hundred μL of diluted *Salmonella* and 100 μL of diluted bacilli were inoculated in 9.8 mL of TSB in order to prepare the mixed culture. For the positive control, 100 μL of diluted sample was inoculated in 9.9 mL of TSB. The cultures were incubated for 24 and 48 h at 37°C, with agitation at 180 rpm. One hundred μL of the mixed cultures and the positive controls was spread on XLT-4 agar (BD Difco, Franklin Lakes, NJ, United States). The plates were incubated at 37°C for 16 h and colonies were then enumerated to assess Salmonella viability.

### Preparation of CFS of the Selected Bacilli Colonies

The selected colonies were inoculated in MRS broth and sub-cultured three times at 37°C for 18 h with agitation at 185 rpm, and the tubes were tilted at 45 degrees during incubation. The third subculture was transferred to an Eppendorf tube (Eppendorf, Hamburg, Germany) and was centrifuged at 16000 *g* for 5 min. The supernatant was filter-sterilized using Millex 0.22 μm non-pyrogenic filters (Merck Millipore Ltd., Co., Cork, Ireland). The pH of the CFS was measured after filter-sterilization and compared against an MRS control.

### Antimicrobial Activity of the Bacilli CFS

A lawn culture from a 1:100 dilution (10^6^ CFU/mL) of overnight grown *M. luteus* was made on tryptic soy agar plates (TSA, BD Difco, Franklin Lakes, NJ, United States). Four 5 mm holes were punched using the wide ed of sterile 200 μL pipette tips, and the agar was removed in order to make four wells on the plate. Then, 100 μL of CFS of either *B. subtilis* KATMIRA1933 or *B. amyloliquefaciens* B-1895 (producing subtilosin A and subtilin respectively) was added to three of the wells. Afterward, 100 μL of MRS broth was added to the fourth well as a negative control. The plates were left under the hood until the diffusion was complete. When the wells had dried, the plates were then incubated at 37°C for 24 h. This experiment was repeated three times.

### *Salmonella* Biofilm Inhibition Assay

Biofilm formation by five strains of *Salmonella* (Thompson, Enteritidis phage type 13a, Enteritidis phage type 4, Typhimurium phage type DT104, Hadar) was tested using flat and round bottom tissue-culture treated Falcon 96 and 48 well plates (Corning, Corning, NY, United States). 1:10 and 1:100 dilutions of 10^6^ CFU/mL were prepared using TSA with 1% glucose. The incubation temperature was 37°C, and the incubation times were 18, 24, and 48 h.

The ability of the bacteriocin containing supernatants to inhibit biofilm formation of *Salmonella* was tested by mixing 100 μL of supernatant with 100 μL of a 1:10 dilution of 10^6^ CFU/mL *Salmonella* in 2x TSB at 2% glucose to make the final concentration 1% in the wells. The 96 well plates were incubated at 37°C for 24 h.

Quantitative analysis of inhibition of biofilm formation was done using the crystal violet (CV) staining assay. After a 24 h incubation period, planktonic cells were removed from each well by a 200 μL pipette. The planktonic cells of the first three wells of each column were transferred to a new 96 well plate for spot plating. The wells were then gently washed three times with 200 μL of phosphate-buffered saline (PBS, Fisher BioReagents, Pittsburg, Pennsylvania, United States). The biofilm was fixed by heating at 60°C for 60 min and stained with CV, according to Borucki et al., 2003. One hundred twenty-five microliter of 0.1% CV was added over the biofilm and left at room temperature (23–25°C) for 20 min. Each well was then rinsed 3–4 times with 200 μl of distilled water and left for 15 min to dry at room temperature. One hundred microliter of 95% (v/v) ethanol in water was added into each well to solubilize the CV-stained biofilm. The plate was then incubated at 4°C for 30 min. After incubation, 100 μL of solution was then transferred from each well into a second 96 well plate. An absorbance measurement was made using an Automated Absorbance reader at 595 nm (Diagnostic Automation, Woodland Hills, CA, United States).

### Enumerating the Cells in Planktonic Cultures by Spot Plating

Spot plating was performed according to Gaudy et al. (1963). Nine hundred microliter of TSB was added to the wells of a sterile deep well plate. One hundred microliter of the planktonic cells, from the 24 h growth of the *Salmonella* with bacilli supernatants, was added to the first well. Seven subsequent 1:10 dilutions were made. Twenty microliter of each diluted solution was spot plated on a TSA plate. The plates were incubated at 37°C for 16 h, and the colonies for each spot were then counted.

### Statistical Analysis

All experiments were conducted a minimum of three times in duplicate. The error bars in the provided figures represent the standard deviations of the data. All calculations were performed in Microsoft Excel, and then the statistical analysis was reshaped with SigmaPlot 11.0 (Systat Software Inc., Chicago, IL, United States). The Student t test and Mann-Whitney Rank Sum Test was also performed using SigmaPlot 11.0.

## Results

### Antibacterial Activity of Single Colonies of *B. subtilis* KATMIRA1933 and *B. amyloliquefaciens* B-1895 Against *M. luteus* ATCC 10240

The colonies with the best inhibitory activity were selected for further experiments. On the third re-culturing, the zone of inhibition of the best performing *B. subtilis* KATMIRA1933 *B.* and *amyloliquefaciens* B-1895 colonies were 11 and 10 mm, respectively.

### *Salmonella* and Bacilli Cross Test

After a 24 h incubation period, no *Salmonella* inhibition was observed with the cross tests. The plates were kept under the hood for an additional 72 h at room temperature. Again, no *Salmonella* inhibition was detected, indicating that there is not direct inhibtion of *Salmonella* growth by the two bacilli and their metabolites.

### *Salmonella* and Bacilli Co-culture

**FIGURE 1:**
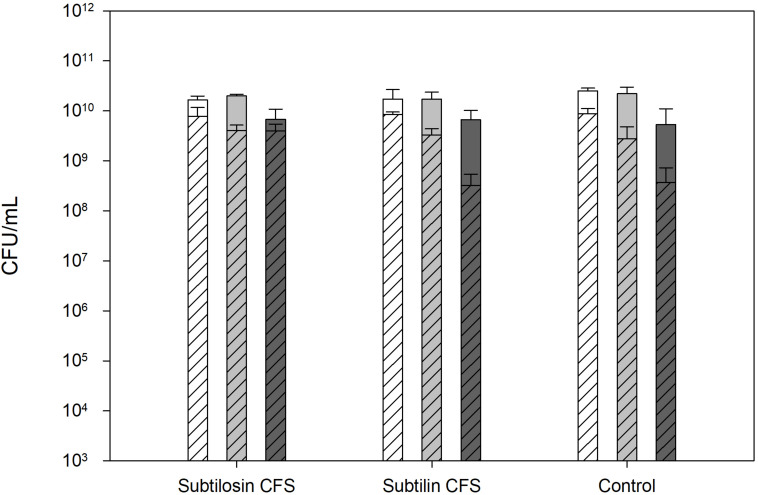
*Salmonella* Hadar (SH 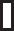), *Salmonella* Enteritidis phage type 4 (SE 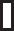), and *Salmonella* Thompson (ST 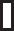) cell count after 24 h (solid bars) and 48 h (diagonal lined bars) exposure to the CFS of *B. subtilis* KATMIRA1933 (subtilosin) and *B. amyloliquefaciens* B-1895 (subtilin).

Figure 1 shows the effects of co-culturing of *B. subtilis* KATMIRA1933 and *B. amyloliquefaciens* B-1895 on the *Salmonella* cell count after a 24 and 48 h incubation period. The CFU/ml of the positive control, the mixture with *B. subtilis* KATMIRA1933, and the mixture with *B. amyloliquefaciens* B-1895 after 24 h was 52.53 × 10^10^, 1.67 × 10^10^, and 1.73 × 10^10^ for *Salmonella* Hadar, 2.20 × 10^10^, 2.00 × 10^10^, and 1.73 × 10^10^ for *Salmonella* Enteritidis phage type 4, and 5.33 × 10^9^, 6.72 × 10^9^, and 6.65 × 10^9^ for *Salmonella* Thompson, respectively. The CFU/ml of the positive control, the mixture with *B. subtilis* KATMIRA1933, and the mixture with the *B. amyloliquefaciens* B-1895 after 48 h was 8.81 × 10^9^, 7.8 × 10^9^, and 8.53 × 10^9^, and 1.73 × 10^10^ for *Salmonella* Hadar, 2.8 × 10^9^, 4.1 × 10^9^, and 3.3 × 10^9^ for *Salmonella* Enteritidis phage type 4, and 3.7 × 10^8^, 4.0 × 10^9^, and 3.2 × 10^8^ for *Salmonella* Thompson, respectively. These results indicate that there is no significant inhibtion of the growth of planktonic *Salmonella* by the bacilli and their metabolites in a liquid environment.

### Antimicrobial Activity of the Bacilli CFS

The average zone of inhibition of the wells containing the *B. subtilis* KATMIRA1933 CFS was 9 mm, and the average zone of inhibition of the wells containing the *B. amyloliquefaciens* B-1895 was 7 mm. The diameter of the wells was 5 mm. No *Bacillus* growth was observed on the plates. The average pH of the tested CFS was 5.83 ± 0.07 and 5.85 ± 0.01 for *B. subtilis* KATMIRA1933 and *B. amyloliquefaciens* B-1895, respectively, as compared to a negative control (MRS medium) at 6.5.

### *Salmonella* Biofilm Formation

*Salmonella* Thompson, Enteritidis phage type 4, and Hadar formed the best biofilm, as determined by relative biomass via crystal violet staining, under the following conditions: 24 h incubation, 1:10 dilution, in round bottom 96 well plates (data not shown).

### Biofilm Inhibition and Enumerations of Planktonic Cells

**FIGURE 2:**
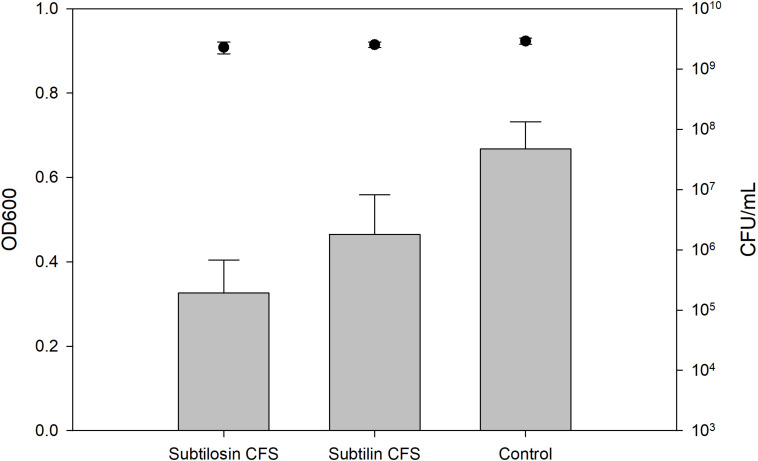
*Salmonella* Hadar biofilm inhibition by *B. subtilis* KATMIRA1933 (subtilosin) and *B. amyloliquefaciens B-1895* (subtilin) (gray bars) and the planktonic cell count (black circles).

**FIGURE 3:**
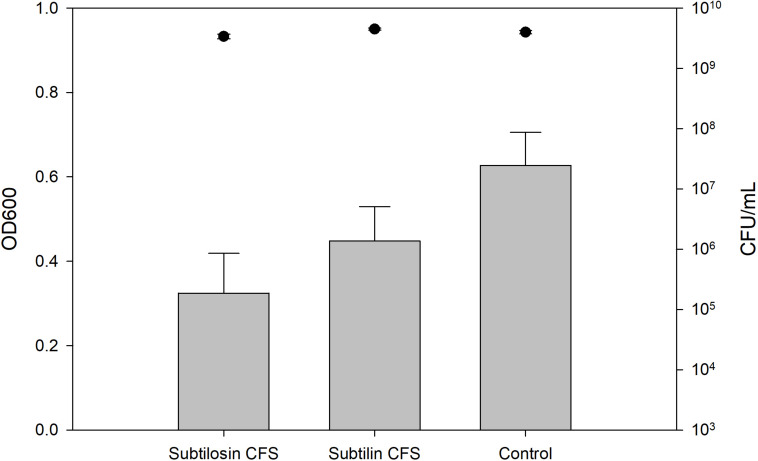
*Salmonella* Enteritidis phage type 4 biofilm inhibition by *B. subtilis* KATMIRA1933 (subtilosin) *B. amyloliquefaciens B-1895* (subtilin) (gray bars) and the planktonic cell count (black circles).

**FIGURE 4:**
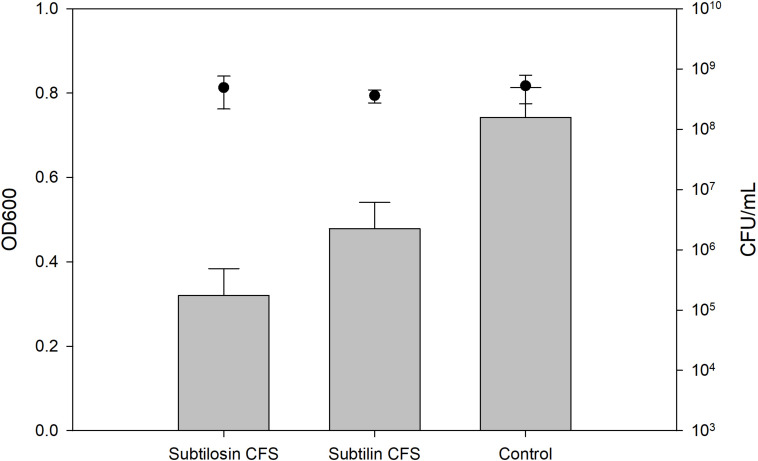
*Salmonella* Thompson biofilm inhibition by *B. subtilis* KATMIRA1933 (subtilosin) and *B. amyloliquefaciens B-1895* (subtilin) (gray bars) and the planktonic cell count (black circles).

Figures 2–4 show the effect of the bacilli CFS on *Salmonella* biofilms and the planktonic cell count for each strain tested. When incubated with *B. subtilis* KATMIRA1933 CFS, biofilm formation of *Salmonella* Hadar, *Salmonella* Enteritidis phage type 4, and *Salmonella* Thompson was inhibited by 51.1% (*P* = 0.001), 48.3% (*P* = 0.001), and 56.9% (*P* = 0.001), respectively. When incubated with *B. amyloliquefaciens* B-1895 CFS, the biofilm formation of *Salmonella* Hadar, *Salmonella* Enteritidis phage type 4, and *Salmonella* Thompson was inhibited by 30.4% (*P* = 0.001), 28.6% (*P* = 0.001), and 35.5% (*P* = 0.001), respectively.

The planktonic cell count of the positive control, incubation with *B. subtilis* KATMIRA1933 CFS, and incubation with the *B. amyloliquefaciens* B-1895 CFS was 2.9 × 10^9^, 2.28 × 10^9^, and 2.52 × 10^9^ for *Salmonella* Hadar, 3.99 × 10^9^, 3.4 × 10^9^, and 4.49 × 10^9^ for *Salmonella* Enteritidis phage type 4, and 5.24 × 10^8^, 4.88 × 10^8^, and 3.6 × 10^8^ for *Salmonella* Thompson. Therefore, the number of planktonic cells was not affected by the treatment.

## Discussion

In this study, it was shown that *B. subtilis* KATMIRA1933 and *B. amyloliquefaciens* B-1895 have potential antimicrobial activity against *Salmonella* through inhibition of biofilm formation ability, which may result in decreased persistence of the pathogen. This points to the rational replacement of antibiotics for the reduction of salmonella in poultry by probiotics whose mode of action is both natural and presents minimal risk of possible complications, particularly compared to the use of antibiotics and the associated risk of increased antibiotic resistance.

Antibiotic resistance in *Salmonella* serotypes was first recognized in the 1960s with the discovery of *Salmonella* resistant to chloramphenicol, and the number and frequency of isolated resistant and multidrug-resistant strains has continued to increase ever since (Montville and Matthews, 2008). Multidrug resistance in *Salmonella* is defined as resistance toward first-generation antibiotics such as ampicillin, chloramphenicol, and trimethoprim-sulfamethoxazole. However, an increasing prevalence of MDR *Salmonella* resistant toward clinically important antimicrobials such as fluoroquinolones and third-generation cephalosporins highlights a worrying trend toward *Salmonella* infections that are increasingly difficult to treat with antibiotics (Jajere, 2019). Lately, there has been growing interest in research focused on utilizing the antimicrobial properties of probiotics against many pathogens, including multidrug-resistant species and strains, with a particular focus on the food, medical, and veterinary industries. In these industries, the uncontrolled and commonplace use of antibiotic regimens greatly increases the chances of resistance development (O’Bryan et al., 2018; Mulani et al., 2019). *B. subtilis* KATMIRA1933 and *B. amyloliquefaciens* B-1895 are two bacilli that have exhibited notable antimicrobial potential and have been demonstrated as beneficial and safe for use as probiotics in poultry (Sutyak et al., 2008; Chistyakov et al., 2015; AlGburi et al., 2016). Poultry species are a common reservoir of Salmonella, and as backyard farming becomes more popular, the number of cases of poultry-to-human transmission of salmonellosis is increasing (McDonagh et al., 2019). This increase highlights the importance of developing novel methods for controlling *Salmonella* infections in poultry that do not carry the same risk of antibiotic resistance development as common antibiotics. Antibiotics are known to alter the composition of microbial flora, often leading to dysbiosis and increased susceptibility of the host to enteric infections by irrational use. The intestinal microbiota plays a key role in maintaining a healthy intestinal environment through various means, such as increasing nutrient availability, modulating the host immune system, and through competitive and antagonistic interactions with pathogens. One of the crucial defense mechanisms of commensal bacteria is to prevent biofilm formation by invasive or opportunistic pathogens (Vuotto et al., 2014). There has been some evidence suggesting that oral consumption of probiotics might decrease the colonization of the GIT by *Salmonella* in chickens. Studies have specifically shown the importance of *Bacillus subtilis* in decreasing the chances of *Salmonella* causing disease (Hayashi et al., 2018).

In order to determine the potential antimicrobial activity of *B. subtilis* KATMIRA1933 and *B. amyloliquefaciens* B-1895 against pathogenic salmonellae, single colonies of these two strains were killed to make sure that any observed inhibition of the common indicator strain, *M. luteus* ATCC 10420, was due to the cellular products of these two strains. This experiment was repeated three times in order to produce colonies with the best antimicrobial activity. The selected colonies were then used for the production of CFS. The antimicrobial activity of CFS was again checked against *M. luteus* ATCC 10420, thereby confirming the presence and activity of bacteriocins in the supernatant. The antimicrobial potential of the of the two bacilli against *Salmonella* was assayed using both a cross test and bacterial co-culture method. Both experiments showed no significant inhibition of *Salmonella* growth due to the presence of the bacilli when grown on solid or liquid media. These results indicate that any observed inhibition of biofilm is likely not due to direct inhibition or killing but is instead due to other mechanisms.

The biofilm formation ability of the three *Salmonella* strains was affected more by *B. subtilis* KATMIRA1933 CFS than *B. amyloliquefaciens* B-1895 CFS. The CFS of *B. subtilis* KATMIRA1933 inhibited biofilm formation of the three salmonellae by approximately 50%, while the CFS of *B. amyloliquefaciens* B-1895 inhibited biofilm formation by about 30%. This degree of inhibition is not dissimilar from the antibiofilm activity of the two strains against MRSA and MSSA, where 25–50% concentrations of CFS were found to inhibit biofilm formation by 45–59% (Algburi et al., 2020a). The antibiofilm activity of the two strains has also been determined for *Proteus mirabilis* isolated from urinary tract infections, where even greater inhibition was seen, with 72–84% inhibition recorded in that study (Algburi et al., 2020b). Interestingly, while the CFS of the two bacilli were found to be effective in inhibiting the biofilm formation in Gram-positive *S. aureus* and *P. mirabilis*, they have now been shown to prevent biofilm formation in Gram-negative *Salmonella*. This is in contrast to the antimicrobial activity of the two probiotics and their CFS against planktonic cells, where they are effective against Gram-positive species, but generally ineffective against Gram-negative species such as *E. coli*, *P. aeruginosa*, and *E. aerogenes* (Huang et al., 2009).

The number of planktonic cells of the three *Salmonella* serovars was not influenced by treatment with either CFS. The 24 and 48 h co-culture experiments also showed that the number of viable cells of the *Salmonella* strains is not significantly altered when grown together with *B. subtilis* KATMIRA1933 or *B. amyloliquefaciens* B-1895. These results, together with previous findings, point toward the possibility of a separate mode of action for the observed anti-biofilm activity that is not necessarily linked to the observed antimicrobial activity of the two strains, nor is it related to direct cell-to-cell contact or co-aggregation.

There are several possible explanations for the observed activity of the CFS, which may be related to the activity of one or more substances produced by the two bacilli, such as subtilosin A and subtilin, or other potential bioactive compounds, such as weak organic acids, enzymes, or bacteriocin-like inhibitory substances (BLIS). Other probiotics have shown that antimicrobial activity against *Salmonella* is linked to a low pH and the production of lactic acid. For example, it has been shown that the antimicrobial activity of *Lactobacillus rhamnosus* GG against *Salmonella typhimurium* is mediated by lactic acid, as confirmed by the antimicrobial activity of *L. rhamnosus* supernatant as compared to a pH matched HCl acid control. However, the activity was found to be pH depended, with a maximum at pH 4.5 and an absence of antimicrobial activity at pH 6.6 (De Keersmaecker et al., 2006). Unlike lactic acid bacteria, sporeforming bacilli produce modest concentrations of lactic acid (10–40 mM), with a moderate decrease of pH from neutral to about 6.5. Noticeably, an engineered strain *B. subtilis* MUR1 is producing up to 2 M of L-lactic acid after 52 h of fermentation (Ohara and Yahata, 1996; Fry et al., 2000).

Under the conditions used in for the cultivation of *B. subtilis* KATMIRA1933 or *B. amyloliquefaciens* B-1895 in this manuscript, there was no significant lactic acid production, as the pH was not controlled for during the 24 h incubation period, with a resulting drop in pH from 6.5 to 5.83 ± 0.07 and 5.85 ± 0.01, respectively. As the antimicrobial activity of lactic acid against *Salmonella* is pH dependent, the effect of lactic acid in this experiment is likely minimal.

Subtilosin has previously been reported to prevent biofilm formation of Gram-variable *G. vaginalis* by inhibiting quorum sensing via a reduction in the production of autoinducer-2 (AI-2). However, in the same study, subtilosin was shown to inhibit biofilm formation of *Listeria monocytogenes* without influencing AI-2 production (AlGburi et al., 2016). Similar results have been found with other bacteriocins, including lactocin AL705, which inhibits biofilm formation at sub-MIC concentrations without reducing the production of an AI-2 like molecule in *L. monocytogenes*, as recognized by a *V. vibrio* reporter strain (Melian et al., 2019). Evidence in the literature on the influence of *luxS/*AI-2 in biofilm formation is inconclusive, and findings cannot be translated from one species to another. A *Haemophilus parasuis* ΔluxS strain had decreased production of AI-2 molecules compared to the wild type and displayed decreased adherence while at the same time having increased abilities to form biofilm *in vitro* (Zhang et al., 2019). In a different study involving an *S. epidermis* ΔluxS mutant, *luxS* was found to repress biofilm formation through a cell-cell signaling mechanism based on autoinducer 2 secretion (Xu et al., 2006). Transcriptomic and phenotypic studies on biofilm formation in a *Salmonella enterica* Serovar Typhimurium Δ*luxS* mutant found that biofilm formation was significantly less in the Δ*luxS* mutant, demonstrating the potential importance of *luxS*/AI-2 for biofilm formation in salmonellae (Jesudhasan et al., 2010). In order to confirm whether the observed biofilm inhibition in this study is linked to inhibition or interference with *luxS*/AI-2 signaling, further experiments with the purified bacteriocins and Δ*luxS Salmonella enterica* serovars will be necessary.

The observed antibiofilm activity may be related to other bacterial communication systems, such as the AI-1 and AI-3 signaling pathways, both of which are present in *Salmonella*. It is also possible that the anti-biofilm activity may be completely unrelated to quorum sensing inhibition and may instead be the consequence of more direct interactions. Both strains produce a variety of proteolytic enzymes, including several subtilisin or peptidase S8 family subtilisin-related serine proteases, that may have potential use in both treating and preventing biofilms (Karlyshev et al., 2014a,b). This family of enzymes is produced by *Bacillus* spp. and able to hydrolyze adhesins that are necessary for proper bacterial aggregation and biofilm formation. Protease treatment has been shown to impact invasion ability, and biofilm formation in *L. monocytogenes* (Longhi et al., 2008), and subtilisin treatment has been used to destroy *Staphylococcus epidermidis* and *Staphylococcus aureus* biofilms (Elchinger et al., 2014). To better understand the mechanisms responsible for the observed biofilm inhibition, the antibiofilm activity of CFS and purified compounds of interest (subtilosin A, subtilin, subtilisin-related serine proteases, lactic acid) should be investigated, and potential quorum sensing activities confirmed through the use of mutant knockouts for the relevant genes in salmonellae of interest.

*B. subtilis* KATMIRA1933 and *B. amyloliquefaciens* B-1895 have been shown to have different modes of activity in poultry. Previous studies reported that when used as probiotics in poultry, they have different impacts on the quality of rooster sperm production, egg production, hatching egg quality, egg hatchability, etc. (Mazanko et al., 2018). Interestingly, when used in combination, these two strains are antagonists. For instance, use of *B. subtilis* KATMIRA1933 alone shows an increase in vitellogenin gene expression levels, resulting in the stabilization of mitochondrial DNA by decreasing relative damage, slowing down reproductive aging, and potentially improving egg-laying ability. The use of a combination of the two species resulted in a decrease in the observed positive effects (Mazanko et al., 2019; Prazdnova et al., 2019). One possible explanation for this phenomenon is that the metabolites of these two probiotic strains have a similar pharmacodynamic mode of action with the same molecular targets.

The main challenge of salmonellosis is in its zoonotic potential, as most animal reservoirs are asymptomatically infected, creating suitable conditions for horizontal and vertical paths of transmission, resulting in the presence of *Salmonella* spp. in up to 65% of birds in a flock (Chlebicz and Śliżewska, 2018). This, in turn, makes it easier for pathogenic bacteria to invade a wide environmental area following human contamination through consumption of livestock and agricultural products (Wiedemann et al., 2014; Kurtz et al., 2017). Salmonellosis a multifactorial infection requiring an integrated approach, and finding appropriate probiotic bacteria may help solve this problem, as they do not require major financial expenseswhen compared to the routine and irrational use of antibiotic therapy and prophylaxis in both the healthcare and agricultural industries (Wang et al., 2019b).

In this paper, it is shown that *B. subtilis* KATMIRA1933 and *B. amyloliquefaciens* B-1895 inhibit biofilm formation of several *Salmonella* serovars, which points to the high potential of using these bacilli strains as probiotics not only as a beneficial feed additive for poultry with the goal of increasing their physiological parameters, but as effective antimicrobial producers and prophylactic agents against *Salmonella* as well. These beneficial properties highlight the high potential of these probiotics for use in poultry resulting in greater economic gain, reduced environmental impact, and improved public health.

## Data Availability Statement

The raw data supporting the conclusions of this article will be made available by the authors, without undue reservation.

## Author Contributions

MT conducted experiments, collected data and conducted the primary data analysis and was responsible for drafting and finalizing the report. All authors contributed equally to the manuscript.

## Conflict of Interest

The authors declare that the research was conducted in the absence of any commercial or financial relationships that could be construed as a potential conflict of interest.

## Footnotes

1 https://www.cdc.gov/nationalsurveillance/pdfs/NationalSalmSurveillOverview_508.pdf

2 https://www.merckmanuals.com/professional/infectious-diseases/gram-negative-bacilli/non-typhoidal-salmonella-infections

3 https://www.cdc.gov/salmonella/infantis-10-18/advice.html
